# What to eat now? Shifts in polar bear diet during the ice-free season in western Hudson Bay

**DOI:** 10.1002/ece3.740

**Published:** 2013-08-28

**Authors:** Linda J Gormezano, Robert F Rockwell

**Affiliations:** Division of Vertebrate Zoology, American Museum of Natural History79th Street and Central Park West, New York, NY, 10024-5192

**Keywords:** Climate change, diet, feces, polar bears, scat, terrestrial, *Ursus maritimus*, western Hudson Bay

## Abstract

Under current climate trends, spring ice breakup in Hudson Bay is advancing rapidly, leaving polar bears (*Ursus maritimus*) less time to hunt seals during the spring when they accumulate the majority of their annual fat reserves. For this reason, foods that polar bears consume during the ice-free season may become increasingly important in alleviating nutritional stress from lost seal hunting opportunities. Defining how the terrestrial diet might have changed since the onset of rapid climate change is an important step in understanding how polar bears may be reacting to climate change. We characterized the current terrestrial diet of polar bears in western Hudson Bay by evaluating the contents of passively sampled scat and comparing it to a similar study conducted 40 years ago. While the two terrestrial diets broadly overlap, polar bears currently appear to be exploiting increasingly abundant resources such as caribou (*Rangifer tarandus*) and snow geese (*Chen caerulescens caerulescens*) and newly available resources such as eggs. This opportunistic shift is similar to the diet mixing strategy common among other Arctic predators and bear species. We discuss whether the observed diet shift is solely a response to a nutritional stress or is an expression of plastic foraging behavior.

## Introduction

Polar bears (*Ursus maritimus*) are the most carnivorous of the Ursids, feeding primarily on ringed seals (*Phoca hispida*) and less frequently on bearded seals (*Erignathus barbatus*) and other marine mammals while sea ice is available for hunting (Stirling and Archibald 1977; Thiemann et al. 2008). Most of this foraging occurs in spring when polar bears accrete the majority of their fat reserves from ringed seals and their newborn pups (Stirling and Øritsland 1995). The ice in western Hudson Bay melts completely by mid- to late July forcing the bears ashore without easy access to their primary prey until freeze-up in the following fall (Gagnon and Gough 2005). While ashore, polar bears are in a negative energy balance (Derocher et al. 1993), reportedly surviving primarily on their fat reserves, although supplementary, terrestrial foods are also consumed when available (e.g., Lunn and Stirling 1985; Derocher et al. 2013). This period onshore is projected to increase as warming trends keep Hudson Bay ice free for progressively longer periods each year (e.g., Stirling and Parkinson 2006). Surviving these extended periods on land without access to seals is believed to be critical to the persistence of polar bears in western Hudson Bay (Molnár et al. 2010).

Polar bears are known to consume various types of terrestrial and marine foods during the ice-free period (hereafter terrestrial or land-based foods). Items reported include marine algae (Harrington 1965), grasses (Koettlitz 1898), berries (Russell 1975), fish (Dyck and Romberg 2007), small mammals (Pedersen 1966; Russell 1975), caribou (*Rangifer tarandus*) (Derocher et al. 2000), seals (Russell 1975), various species of waterfowl and their eggs (e.g., Stempniewicz 1993; Drent and Prop 2008; Rockwell and Gormezano 2009), and willow ptarmigan (*Lagopus lagopus*) (Miller and Woolridge 1983).

Despite these observations, some of which date back to the late 1800s (Koettlitz 1898), polar bears are often referred to as “fasting” while ashore (e.g., Amstrup et al. 2007; Molnár et al. 2010; Robbins et al. 2012). Although the term may apply to some polar bears, extension to the majority of the western Hudson Bay population seems inappropriate given multiple observations to the contrary (see above), and the inherent limitations of behavioral and physiological studies (Knudsen 1978; Latour 1981; Ramsay and Hobson 1991; Hobson et al. 2009) that are often used to justify the term's use. For example, observational studies may only offer a snapshot of behavior for discrete periods (Knudsen 1978; Latour 1981) and coastal or inland sampling may preclude certain demographic groups because they tend to spatially segregate once ashore (Latour 1981; Derocher and Stirling 1990). Physiological studies, such as stable carbon isotopes and fatty acid signatures offer a more integrated assessment of the diet but are fraught with inconsistencies. For example, stable carbon isotopes can give variable results depending on the tissue examined (Ramsay and Hobson 1991; Hobson et al. 2009) and the mixing of marine and terrestrial signatures of foods polar bears commonly consume on land (e.g., marine algae, waterfowl feeding in salt marshes; McMillan et al. 1980; Hobson et al. 2011). Fatty acid signatures can vary by individual depending on differential accumulations and deficits (Pond et al. 1992; Grahl-Nielsen et al. 2003).

The direct analysis of passively sampled scat offers several advantages for determining dietary details on the extent and pattern of land-based foraging by polar bears. Scats deposited reflect foods consumed over longer spans (i.e., spring, summer, or fall), through various diurnal cycles, and during weather changes in which periods of active foraging may fluctuate. Although exact numbers and sexes of polar bears sampled cannot be assessed from scat in the absence of genetic analyses, collection of scats over a large geographic extent increases the chances of sampling from different sex and age groups and from different individual polar bears given their tendency to move relatively little once ashore (Derocher and Stirling 1990; Parks et al. 2006). While exploring the nutritional and energetic value of terrestrial food is beyond the scope of this study, we use scat analysis to examine the land-based diet of polar bears across a large portion of the terrestrial habitat used during the ice-free period in western Hudson Bay.

Reports of polar bears exploiting land-based prey have become more common in recent years (e.g., Derocher et al. 2000; Drent and Prop 2008; Rockwell and Gormezano 2009; Iles et al. 2013). For example, consumption of eggs and young from nesting colonies of waterfowl across the Arctic is increasingly pervasive, and predation on larger land mammals, such as caribou, had been reported (Derocher et al. 2000). Although categorized as specialists that primarily hunt seals on the ice (Derocher et al. 2004; Amstrup et al. 2007), polar bears have been observed walking, running, and even climbing cliffs (Smith et al. 2010) on land to pursue alternate prey. Like other bear species, polar bears may well be opportunists, pursuing the most readily available food source (Lunn and Stirling 1985; Beckmann and Berger 2003; Thiemann et al. 2008). It remains unclear whether exploiting these alternate foods (behavioral shifts) is mainly a response to nutritional stress or simply a typical Ursid response to a changing food supply.

To better understand how polar bears may be reacting to climate change or other environmental factors, we first created a comprehensive inventory of the current polar bear diet across their terrestrial range in western Hudson Bay by analyzing passively collected scat. Second, to identify any dietary shifts during the ice-free season that may have occurred since the recent onset of rapid climate changes we compared our data to a similar scat-based diet study performed in the Hudson Bay Lowlands 40 years earlier by Russell (1975). In parallel with this comparison, we compared the average 50% breakup dates during this and Russell's diet study as an index of climate-related environmental change between the two time periods. Finally, we explore other possible bases for the observed shifts in land-based foraging we document and discuss the implications they have for polar bears' ability to persist in the face of reduced ice conditions that limit their time to hunt seals.

## Material and Methods

### Study area

**Figure 1:**
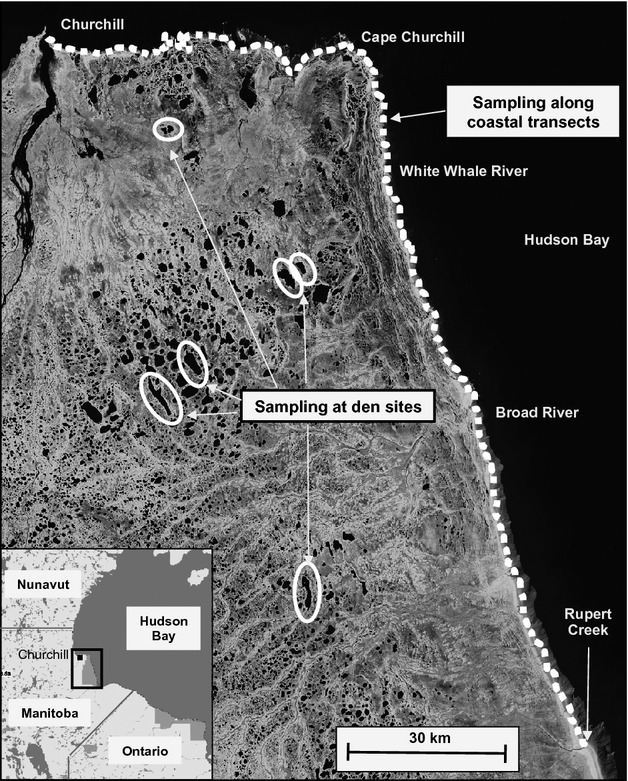
Polar bear scat was collected along the coast of western Hudson Bay from the town of Churchill, Manitoba, to Rupert Creek. Scat was also collected near maternity dens at six inland sites. Collections were made from 2006 through 2008.

Scat sampling occurred along 160 km of coastline and adjacent inland areas of what is now termed the Cape Churchill Peninsula (Rockwell et al. 2011) where polar bears are known to occur during the ice-free period in western Hudson Bay (Derocher and Stirling 1990). Coastal areas within the study area extended from the town of Churchill, Manitoba (58°46'N, 94°12'W), east to Cape Churchill (58°47'N, 93°15'W) and south to Rupert Creek (57°50'N, 92°44'W). We also collected samples from six separate denning areas southeast of Churchill and inland of the coastline to 93°51W' (Fig. 1). By including both coastal and inland denning habitat we can provide a more complete inventory of the land-based diet of all demographic groups that differentially use this region (Latour 1981; Derocher and Stirling 1990).

The coastline south of Cape Churchill is largely flat with poor drainage, characterized by salt marsh interspersed, as one moves inland, with successively older relict beach ridges that run parallel to the coast (Dredge 1992). The vegetation along that section of coastline, as well as the better drained coastline from Churchill to Cape Churchill, is dominated by sedges (i.e., *Carex* spp.), grasses (e.g., *Puccinellia phryganodes*, *Dupontia fisheri*), and herbs (e.g., *Primula egaliksensis*, *Parnassia palustris*) with interspersed woody shrubs including willow (*Salix* spp.), birch (*Betula glandulosa*), and *Rhododendron lapponicum* (Ritchie 1960).

The inland denning sites and the more inland areas near Churchill, Manitoba, are in the ecotone between boreal forest and low Arctic tundra. The area is a mosaic of vegetation communities including open canopies of white spruce (*Picea glauca*), black spruce (*P. mariana*), and tamarack (*Larix laricina*). Forested areas are interspersed with sedge meadows (primarily *Carex aquatilis*), upland lichen-heaths bogs with *Vaccinium uliginosum*, *Cladina rangiferina,* and *Sphagnum* spp., and fens with shrubby vegetation such as willow and birch (Ritchie 1960). Polar bear dens are often dug into frozen peat banks of rivers or lakes at the base of black spruce trees or beneath permafrost hummocks (Clark et al. 1997).

Onshore movement of polar bears in western Hudson Bay coincides with the breakup of sea ice, and an algorithm based on 50% spring ice cover has often been used as a reliable predictor of arrival date (Stirling and Parkinson 2006). Using this approach, Lunn (2008) predicted that polar bears arrived onshore shortly after 24 June, 22 June, and 28 June in 2006, 2007, and 2008, respectively. We used 24 June, the mean breakup date, as an index of current environmental conditions and compared it to the mean breakup date during Russell's (1975) study as a means to compare dietary differences coincident with changes in environmental conditions. From Lunn (2008) we used the earliest 5-year period in that data set (1971–1975) and projected the mean breakup date for 1968–1969 using a linear relationship previously developed by Rockwell and Gormezano (2009).

### Fecal collection

Fecal piles were found using a trained detection dog along 31 linear coastal transects and in the vicinity of inland dens in the six denning areas from 2006 through 2008. The numbers of scats collected each year was not fixed a priori. Transects were 1–3 kilometers long and were parallel to the coastline. Coastal transects between the town of Churchill, Manitoba, and the White Whale River were walked between 25 May and 7 August, and coastal transects from Cape Churchill to Rupert Creek were walked between 14 July and 11 August. Upland habitat in the vicinity of inland dens was searched between 30 May and 17 June. The collection team was transported to and from all collecting sites by helicopter (except those accessible by truck near Churchill) and the team consisted of the coauthors, the detection dog, and, when possible, an additional armed polar bear warden.

Intact scat piles were placed in plastic bags and stored frozen at −20°C until analyzed. Date, geographic coordinates, substrate, and relative freshness were recorded for each sample. Intact piles of all ages were collected. Scat piles were often found to be clumped along a transect or near a denning site. To reduce potential bias resulting from multiple scat piles being deposited by a single individual, we did not use all the samples collected from clumped points along each of our 31 transects for these diet analyses. We also subsampled across the entire collection so that the scat piles analyzed for diet were representative of the relative frequencies and geographic extent of the sampled areas. Although the actual number of polar bears depositing the sampled scats remains unknown, we assume from the size and geographic extent of our sampling and the facts that once ashore polar bears segregate and move little once ashore (Derocher and Stirling 1990; Parks et al. 2006) that our samples are representative of the land-based diet of those polar bears that do forage on the Cape Churchill Peninsula during the ice-free period.

### Fecal analysis

Entire scats were defrosted, broken apart, and examined for plant and animal remains using flame-sterilized forceps. To preserve specimens for future genetic tests to identify individual bears, we did not use washing techniques (e.g., Russell 1971; Hewitt and Robbins 1996). Multiple bone, hair, and feather samples believed to represent individual prey animals were removed from each pile. These specimens were cleaned by soaking and gently rubbing in a bowl with water and mild soap and assigned to species or the finest taxonomic level possible. Taxonomic determinations were made independently from each hair, bone, and feather specimen in the same pile to minimize assignment bias because animals of different species were often found in the same pile. Unique plant items were removed from scats and also identified to the lowest taxonomic level. Garbage constituted all items from anthropogenic sources (e.g., plastic, paper, apples). We considered food items (other than polar bear, see below) to occur in a scat if any amount of that food item, regardless of volume, was present. For consistency, all analyses were performed by the lead author.

Based on the morphology of bone fragments, the type and source taxa were identified using museum skeletons, reference keys (Wolniewicz 2001, 2004; Post 2005), and expert opinion (N. Duncan and A. Rodriguez, pers. comm.). If specimens could not be identified beyond “bird” or “mammal,” they were marked as “indeterminable” and only included in statistical analyses where the pooled, higher taxonomic groups (i.e., birds, mammals) were used. Bones classified no finer than “animal” were only included in summary statistics of major food categories (e.g., vegetation, animals).

Hairs were identified where possible by comparison to a reference collection (obtained from harvested animals in the study area) using morphological features such as color, pattern, length, and texture. Hairs that could not be identified this way were mounted on 3 × 1″ glass slides with Flo-Texx® mounting medium (Lerner Laboratories, Delray Beach, FL), covered with 22-mm glass slide covers and examined under 10, 20, and 40× magnification with a compound light microscope. Cuticle-scale patterns and the shape and presence of the medulla were compared to the reference collection, museum specimens, and a key (Brunner and Coman 1974). Lack of observable structural differences for some samples limited identification to genus (e.g., *Lepus* spp.), family (e.g., Phocidae, Cricetidae), or order (e.g., Cetacea). Unidentifiable hairs were classified as belonging to “indeterminable mammals.” Most scats contained polar bear hair which was likely ingested during grooming. Evidence of cannibalism, however, was distinguished from grooming by the larger volume of hair, presence of flesh, bone, and a distinct smell.

Bird feathers from scat were identified by comparing shape, size, and color patterns with museum specimens. We also used barbule node patterns of feathers of unknown birds, in comparison with reference slides and published guides, to make taxonomic identification (Dove and Agreda 2007; C. Dove, pers. comm.). Downy barbs from the plumulaceous region were removed from both sides of the vanes with forceps, elongated and mounted in a similar manner to hairs. The presence, position, and density of nodes on barbules viewed at 10–40× magnification using a compound light microscope were used to identify birds to the lowest taxonomic level.

In addition to these morphological characteristics, we used knowledge of which birds overlap polar bears onshore in western Hudson Bay in making some final taxonomic determinations (Rockwell et al. 2009). For example, individual feathers and node patterns of Brant and Canada Geese (*Branta bernicla* and *B. canadensis*, respectively) appear similar, but only Canada Geese nest and molt in the region when polar bears are present and at a time when they are most vulnerable to predation. Consequently, feathers with a morphological match to both species were classified as Canada geese.

Plants and fungi from scats were identified using keys (Johnson 1987; Marles et al. 2000); however, due to the variety encountered and time constraints, we pooled occurrences of samples into broad taxonomic groups. These included marine algae (e.g., *Fucus* spp., *Laminaria* spp.), berries (e.g., *Vaccinium uliginosum*, *Empetrum nigrum*), lichens (e.g., *Cladina stellaris)*, mosses (e.g., *Sphagnum fuscum*), and mushrooms (*Lycoperdon* and *Bovista* spp.). Due to the high occurrence of Lyme grass (*Leymus arenarius*) shafts and their protein-rich seed heads (Facciola 1998) in scat and observations of bears targeting just seed heads (Gormezano and Rockwell, in review) that emerge in July (Johnson 1987), we separated “Lyme grass” (shafts and/or seed heads) and “seed heads” (only seed heads, no shafts) into different categories for some analyses. We pooled all other grass species, such as *Festuca brachyphylla*, into “other grasses.” Leaves and stems of shrubs and woody plants (e.g., *Salix planifolia*, *B. glandulosa*) were not quantified in our study because they consistently comprised <1% of individual scat piles and we assumed that they were either accidentally ingested or picked up from the substrate during collection.

We compared the contents of polar bear scats to those reported in Russell (1971), who used different techniques to identify food items. These included soaking previously dried scats, washing them through a series of screens and examining the contents using both macroscopic and microscopic techniques (Russell 1971). Russell's method of washing entire piles may have resulted in identification of more food items, thus findings of lower frequencies in the current diet may be due to lower consumption of those foods or missing those foods during examination. Conversely, finding more items in the current diet would support higher consumption of those foods and be less likely the result of sampling error. Furthermore, we took advantage of more recent advances in microscopic techniques to identify bird remains that were not available during Russell's study (e.g., Dove and Agreda 2007) and which may have contributed to differences in the number of specific taxa identified between the two studies.

It is worth noting that scat analysis has inherent advantages and limitations that affected both studies (Reynolds and Aebischer 1991). For example, although scat collections were noninvasive, eliminating impacts of capture and handling, exact information on individual animals and times of deposition could only be inferred. Furthermore, due to differential digestion, foods possessing less digestible parts (e.g., fibrous plants, fur, bone) were easier to identify, and thus may be overly represented compared to highly digestible foods (e.g., seal and whale blubber, fish; Best 1985; Hewitt and Robbins 1996).

### Statistical analysis

We examined the diet of polar bears using 14 inclusive groups of food items with each group having at least five occurrences of all the included taxa. These groups were polar bear, seal, caribou, rodents (i.e., muskrats [*Ondatra zibethicus*], meadow voles [*Microtus pennsylvanicus*], collared or bog lemmings [*Dicrostonyx richardsoni* and *Synaptomys cooperi*]), birds, eggs, Lyme grass shafts, Lyme grass seed heads, other grasses, marine algae, berries, mosses, mushrooms, and garbage. Although the seed heads of Lyme grass originate from the same plant as the shafts, their occurrences within scat piles are independent (see below).

Both the (1) raw frequencies (number of times each food item was found) and (2) scat occurrences (the number of scats with a food item) were used in statistical analyses. We use the percentages of these (relative to their appropriate sum) for ease of presentation in some cases. The raw frequencies and the number of scat occurrences are the same value unless multiple items from the same category occur in a scat pile (i.e., two birds in one scat pile). Multiple items were only counted for animals when evidence was conclusive (e.g., three bird feet) and were not counted for plants and fungi. Depending on the analysis, we conflated food items into inclusive taxonomic groups (e.g., birds vs. mammals, animals vs. plants), which allowed us either to reduce problems of small numbers within group sample sizes or to address broader and more general questions. Because we did not determine digestibility of different foods, we did not include volumetric measures to infer the energetic contribution of different foods in the polar bear diet (Reynolds and Aebischer 1991).

Piles of scat often contained more than one food item, reflecting that bears may eat more than one item at a time or one scat pile may represent multiple feeding sessions. Because we were interested in the individual items consumed, we used the raw frequencies of items instead of the scat occurrences as the unit of measure in statistical analyses. To justify this approach, however, we first needed to determine whether food items occurred independently across scat piles. Using occurrences of pairs of food items in scat (co-occurrences), we conducted multiple 2 × 2 log-likelihood chi-square tests (Zar 1999) to evaluate whether the frequencies of individual food items occur independently from all others across scat piles. Significance of these pairwise and subsequent multiple comparison tests was evaluated using a sequential Bonferroni approach (Holm 1979) to reduce inflation of our overall alpha error rate.

### Comparison of diet changes over time

We compared the distribution of food items found in our 642 scat piles sampled from 2006 to 2008 to those found from 1968 to 1969 in 212 scat piles collected in three areas along the west and south coast of Hudson Bay (Cape Churchill, West Pen Island and Cape Henrietta Maria) by Russell (1971, 1975). He pooled the data on food items found in the scat over the three areas and 41% of his samples were from the Cape Churchill area, which is common with our study. Although the exact extent of his sampling in the Cape Churchill area is not clear, it is known that most researchers worked out of the “Cape Churchill camp” (now referred to as Nester 1), located 14 km south of Cape Churchill. Sampling from the camp typically covered a 76 km range from the Cape (58°47'N, 93°15'W) to the Broad River (58°07'N, 92°51'W; L. Vergnano, pers. comm.). His other sites are south and east. The difference in geographic coverage leads to an asymmetrical problem for inferences from comparisons between the two studies. If we fail to find one of the food items he reported or find that an item has decreased in frequency, we can draw inferences regarding changes in food use only by assuming that his pooled proportions for particular food items are representative of the Cape Churchill area. By contrast, however, if we find a new food item or an increase in the proportion of an item, we can reasonably conclude that the item is now being used or being used more in the Cape Churchill area since the 1960s.

We used raw frequencies from both studies in our statistical analyses and percent frequencies and percent scat occurrences in depicting the results. Raw frequencies for each of Russell's food items were obtained from Table 7 (p. 30) in Russell (1971) and pooled across volume categories. Because Russell's sample sizes were smaller, we combined food items into nine inclusive groups with each group having at least five occurrences of all included taxa. The groups were mammals, birds (including eggs), Lyme grass, other grasses, marine algae, berries, mosses, mushrooms, and garbage (referred to as “debris” by Russell). Russell did not separate out parts of the Lyme grass plant so all references to “Lyme grass” include a composite of shafts and/or seed heads, as it does in our study. Other food items, such as cetaceans, lagomorphs, insects, marine invertebrates, fish, lichens, club mosses, horsetails, rushes, and sedges, were found in very low frequencies or not specifically classified in either study so were excluded from chi-square tests. The data from Russell (1971, 1975) were collected from coastal areas, whereas our data were from both coastal and inland areas. Before the comparison with Russell's data, we used 2 × 9 log-likelihood chi-square test to evaluate differences in the frequencies of nine major food items between coast and inland areas during our study. Based on the results, we excluded our inland data from all statistical comparisons with Russell's data.

Pooling major food groups, we used a 2 × 3 log-likelihood chi-square test to evaluate whether there was a difference in the proportions of animals, vegetation, or garbage consumed by polar bears between the late 1960s and present. We then compared the proportions and 95% confidence limits to determine which category was responsible for the observed differences. On the basis of the relationship between the binomial and *F* distributions, we calculated exact 95% upper and lower confidence limits for each proportion and used single and double harmonic interpolation to calculate *F* critical values for large values of *n* (Zar 1999). To determine if there were shifts in the types of foods consumed within these broader categories, we used a 2 × 9 log-likelihood chi-square test to evaluate whether there were differences in the frequencies of nine inclusive food groups (described above) consumed between time periods. On the basis of the results of this test, we compared the proportions and 95% confidence limits of food item frequencies to assess which individual groups differed. For this comparison, we further broke down the “mammal” category into polar bears, seals, rodents, and caribou and “birds” was separated into birds and eggs.

Using all animal taxa identified to the finest level possible in either study (including those excluded from the chi-square analyses, see above) along with the major plant categories described above (with the addition of lichens), we used a Mann–Whitney test to further compare the two diets. The Mann–Whitney test is a nonparametric test that uses the degree of variability or dispersion between two groups to evaluate whether the rank order of the observed frequencies of food items is derived from the same diet (Zar 1999).

## Results

We collected a total of 1262 scats and analyzed 642 of them; 219, 248, and 175 in 2006, 2007, and 2008, respectively (Table 1). Of these, 593 scats were collected from coastal areas and 49 from inland areas. Nearly one-third (29.0%) of all scats contained bird and/or egg remains, the majority of which were snow geese (43.1% of bird remains) and Canada Geese (9.7% of bird remains). Eggs occurred in 4.4% of scats. The most common mammals were caribou (10.1%), seal (most likely *P. hispida*) (6.5%), and polar bear (from cannibalism, not grooming) (5.1%), with small mammals (i.e., rodents, Arctic or snow-shoe hares [*Lepus arcticus* and *L. americanus*]) occurring in lower frequencies (<1.0%). Grasses (61.7%; mainly Lyme grass, 57.0%) and various species of marine algae (46.1%) were the primary forms of vegetation. Other common food items include mosses, puffball mushrooms, and berries, occurring in 13.6%, 8.9%, and 8.7% of scats, respectively.

**Table 1:** The frequencies of food items in 642 polar bear scats from western Hudson Bay 2006-2008

	Raw frequencies*		Scat occurrences
		
Taxa	*n*	%	%
*Birds*			
Aves, indeterminable	45	3.3	7.0
Anatidae, indeterminable	14	1.0	2.2
Anserinae, indeterminable	6	0.4	0.9
Anser caerulescens caerulescens	80	5.9	12.5
Branta Canadensis	18	1.3	2.8
Anatinae, indeterminable	2	0.1	0.3
Anas rubripes	1	0.1	0.2
Anas crecca	1	0.1	0.2
Anas acuta	1	0.1	0.2
Merginae			
Mergus serrator	3	0.2	0.5
Somateria mollissima	2	0.1	0.3
Melanitta perspicillata	1	0.1	0.2
Galliformes, *Lagopus lagopus*	3	0.2	0.5
Passeriformes*, Plectrophenax nivalis*	1	0.1	0.2
Charadriiformes, indeterminable	1	0.1	0.2
*Limnodromus griseus*	1	0.1	0.2
Egg shell/hatching membrane	28	2.1	4.4
Aves – total	208	15.3	29.0
*Mammals*			
Mammalia, indeterminable	6	0.4	0.9
Phocidae	42	3.1	6.5
Ursidae, *Ursus maritimus*	33	2.4	5.1
Cervidae, *Rangifer tarandus*	65	4.8	10.1
Cricetidae, indeterminable	3	0.2	0.5
Ondatra zibethicus	3	0.2	0.5
Microtus pennsylvanicus	1	0.1	0.2
Lemmini	1	0.1	0.2
Cetacea	1	0.1	0.2
Lagomorpha, *Lepus spp*.	2	0.1	0.3
Mammalia – total	157	11.6	22.0
Animal (Mammal or Bird), indeterminable	11	0.8	1.7
*Marine invertebrates*			
Asteroidea (sea stars)	1	0.1	0.2
Bivalvia, *Mytilus edulis*	4	0.3	0.6
Fish	2	0.1	0.3
Insects	3	0.2	0.5
Grasses			
*Leymus arenarius* (43 had seed heads)	366	27.0	57.0
Other grasses	67	4.9	10.4
Grasses – total	433	31.9	61.7
*Mushrooms*			
*Lycoperdon pyriforme* or *L. perlatum*	57	4.2	8.9
Marine algae	296	21.8	46.1
Mosses	87	6.4	13.6
Berries	56	4.1	8.7
Lichens	1	0.1	0.2
Garbage**	41	3.0	6.4

Data are presented as (1) the number of times each food item was found (raw frequencies), (2) raw frequencies/total frequencies (*n* = 1357) of all food items (percent frequencies), and (3) the number of scats with a food item/total number of scats (percent scat occurrences).

* the number of scat occurrences is excluded because it is the same value as the raw frequencies for all food items except birds. We were able to identify multiple birds in seven of 180 (3.9%) scats with birds.

** includes apple peel, aluminum foil, cantaloupe seed, cardboard, corn kernel, chicken bone, cigarette butt, duct tape, foam rubber, glass, paint chips, paper, plastic, string, tomato seed, watch band, and wood chips/sticks.

No pairs of food items in scat piles showed significant patterns of co-occurrence at our adjusted alpha error level, and we therefore consider food items to occur independently in scats. This lack of co-occurrence justifies the use of the raw frequencies of food items as a unit of measure in subsequent statistical tests rather than the number of scats containing each item. Perhaps not surprisingly, marine algae and berries were observed together less often than expected (*G* = 6.31, df = 1, *P* = 0.013), although the result did not reach the adjusted alpha level (14 tests; α = 0.0035) required to avoid error inflation.

### Comparison of diet changes over time

**Figure 2:**
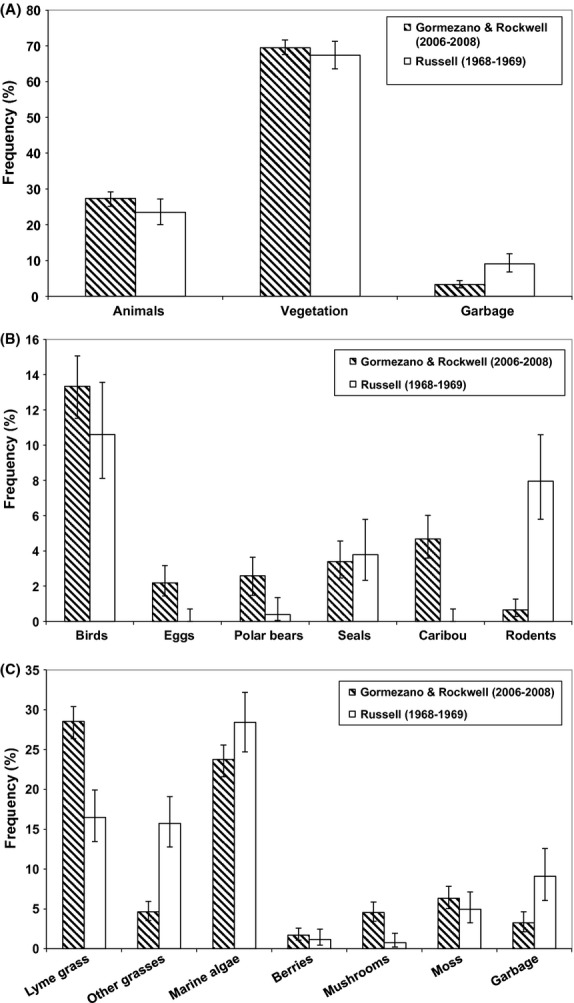
The percent frequencies of some food items found in scat along the coast of western Hudson Bay differed between collections made in 1968–1969 and 2006–2008. Analytical 95% confidence intervals are indicated for each. Note the *y*-axis scale differences in the depictions for (A) pooled categories (animals, vegetation, and garbage) and individual (B) animal and (C) plant, fungi, and garbage food items.

We compared 593 scats (1237 occurrences) of our coastal data with 212 scats (528 occurrences) from Russell's study to examine polar bear diet changes over time. We found a shift in the frequencies of major food categories (animals, vegetation, garbage) (*G* = 25.54, df = 2, *P* < 0.0001). This result was due to a larger proportion of animals (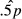 = 27.32, CI = 25.13–29.18 vs. 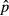 = 23.48, CI = 20.00–27.16) and less garbage (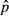 = 3.23, CI = 2.11–4.61 vs. 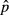 = 9.09, CI = 6.05–12.58) in scats in our study compared to Russell's study (Fig. 2A). Within these major food categories, there were differences in the frequencies of nine major food items (birds, mammals, Lyme grass, other grasses, marine algae, berries, mushrooms, moss, and garbage; *G* = 130.31, df = 8, *P* < 0.0001). The two diets (historic vs. current) also differ in the rank order of items (Mann–Whitney test: *U* = 317; *P* = 0.015).

Among animals, rodents occurred considerably more frequently in Russell's study (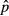 = 7.95, CI = 5.80–10.59) than in ours (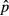 = 0.65, CI = 0.28–1.27), but we observed more polar bear remains (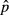 = 2.59, CI = 1.48–3.63 vs. 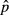 = 0.38, CI = 0.05–1.36). Russell did not detect any caribou, whereas caribou was the most common mammal found in our study (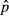 = 4.69, CI = 3.58–6.02). There was no significant difference in the frequencies of seals or birds, but we found eggs in scats (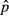 = 2.18, CI = 1.44–3.16), whereas Russell did not (Fig. 2B).

Observed differences in vegetation were due to higher proportions of Lyme grass (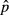 = 28.54, CI = 26.35–30.40 vs. 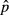 = 16.48, CI = 13.46–19.90) and mushrooms (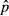 = 4.53, CI = 3.44–5.85 vs. 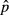 = 0.76, CI = 0.21–1.93), but lower proportions of other grasses (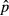 = 4.61, CI = 3.51–5.93 vs. 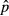 = 15.72, CI = 12.75–19.09) and marine algae (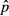 = 23.77, CI = 21.57–25.59 vs. 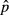 = 28.41, CI = 24.70–32.18) were observed in our study. There were no significant differences in the proportions of berries and mosses (Fig. 2C). These data are summarized as both percent frequencies and percent scat occurrences for comparison in Table 2.

**Table 2:** Comparison of food items in polar bear scats from coastal areas of western Hudson Bay, Manitoba, (2006-2008) and Cape Churchill, Cape Henrietta Maria, and the west Pen Island (1968-1969)

	Gormezano & Rockwell (2006-2008)			Russell (1968-1969)		
		
	Raw frequencies		Scat occurrences	Raw frequencies		Scat occurrences
				
Taxa	*n*	%	%	*n*	%	%
Birds						
Aves	122	9.9	18.0	4	0.8	1.9
Aves – unidentified	43	3.5	7.3	52	9.8	24.5
Egg shell/hatching membrane	27	2.2	4.6	0	0.0	0.0
Aves total + eggs	192	15.5	28.8	56	10.6	26.4
Mammals						
Phocidae	42	3.4	7.1	20	3.8	9.4
Ursidae, *Ursus maritimus*	32	2.6	5.4	2	0.4	0.9
Cervidae, *Rangifer tarandus*	58	4.7	9.8	0	0.0	0.0
Cricetidae	8	0.6	1.3	42	8.0	21.7
Mammalia – unidentified	6	0.5	1.0	4	0.7	1.9
Mammalia – total	146	11.8	24.6	68	12.9	32.1
Grasses						
Leymus arenarius	353	28.5	59.5	87	16.5	41.0
Other grasses	57	4.6	9.6	83	15.7	39.2
Grasses – total	410	33.1	63.1	170	32.2	80.2
Marine algae	294	23.8	49.6	150	28.4	70.8
Berries	21	1.7	3.5	6	1.1	2.8
Mushrooms	56	4.5	9.4	4	0.8	1.9
Mosses	78	6.3	13.2	26	4.9	12.3
Garbage	40	3.2	6.7	48	9.1	17.0

Data are presented as the percent frequencies of all food items (*n* = 1237, *n* = 528) and the percent scat occurrences (*n* = 593, *n =* 212*)* for the current and past polar bear diets, respectively.

Coincident with these dietary changes, we estimated the mean breakup date during Russell's study (1968–1968) to have been 17 July, which is 22 days later than the mean breakup date for this study (2006–2008).

## Discussion

If the trend toward earlier spring ice breakup in Hudson Bay continues, polar bears will spend more time onshore during summer, making any foods consumed during this period increasingly important for the bears' persistence. Their current land-based diet is diverse, consisting of many plants and animals, often consumed together in various combinations. Numerous scats were collected across the entire Cape Churchill Peninsula, from both coastal and inland areas. Given the spatial extent of our sampling, and the propensity for bears to segregate (Latour 1981; Derocher and Stirling 1990) and to move relatively little once ashore (Parks et al. 2006), we assume our results reasonably reflect the land-based diet of those polar bears that do forage on the Cape Churchill Peninsula during the ice-free period. However, consistent with behavioral observations we have made (Iles et al. 2013; Gormezano and Rockwell, in review) and foraging reports by others (e.g., Dyck and Romberg 2007; Smith et al. 2010), it appears that a number of polar bears do not abstain from eating during the ice-free period. Continued use of the term fasting to describe the behavior of polar bears in general during this period (e.g., Stirling and Derocher 2012) seems rather misleading.

Many foods polar bears are consuming have not changed since the 1960s on the Cape Churchill Peninsula, but we did find new foods and marked changes in the frequency of others. The overall proportion of animals in the diet has increased, whereas the proportion of vegetation has not changed. Caribou and eggs are now present in the diet, the proportion of polar bear remains has increased and that of small mammals has decreased. We also identified more species of birds (11 vs. 1), the majority of which were lesser snow geese. Most scats contained at least one type of vegetation and there were only minor shifts in the types consumed. We also found less garbage in scats than was found in the 1960s (Russell 1975). In the following, we discuss various habitat and environmental changes that occurred during the ensuing 40 years coincident with observed diet changes, including a 22-day advance in the date of sea ice breakup and the closing of the Churchill dump.

**Figure 3:**
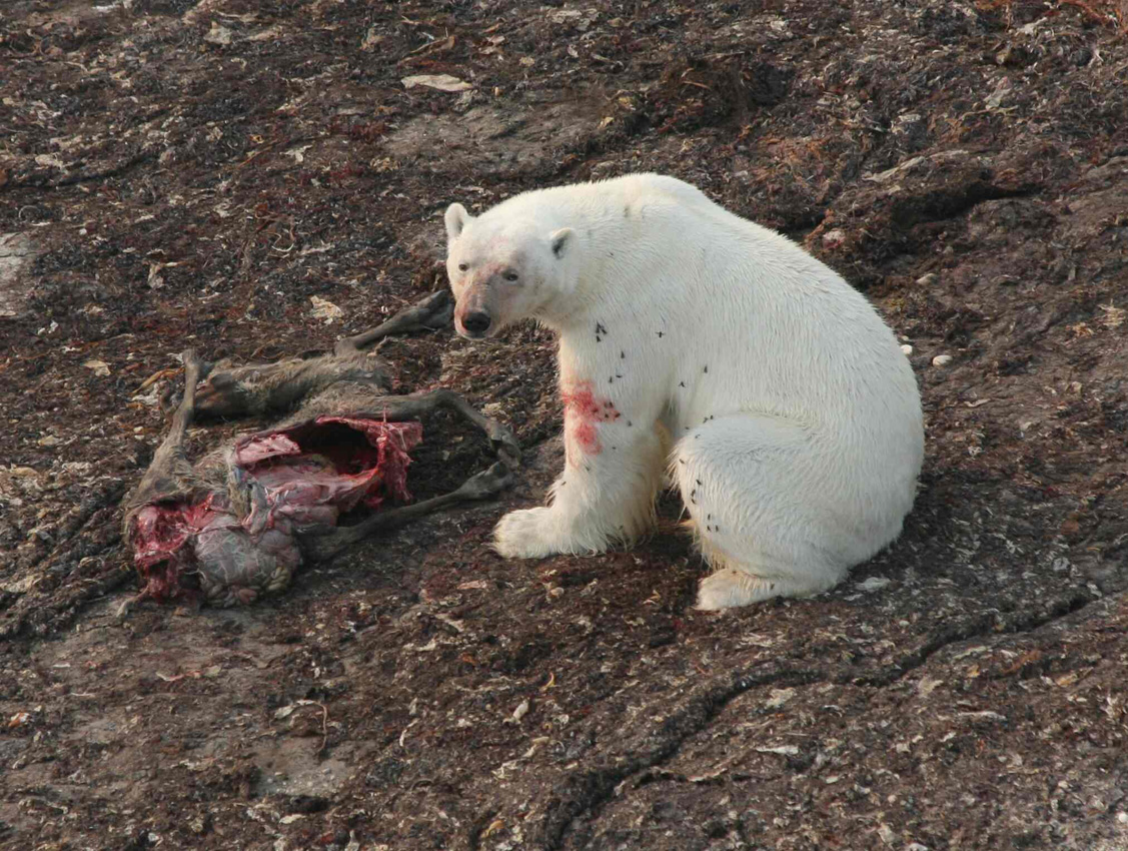
A polar bear looks up from the recently killed caribou it was eating at Keyask Island (58.16958°N 92.85194°W) on July 26, 2010. Photograph by R. F. Rockwell.

Russell did not report caribou or snow geese in polar bear fecal samples collected along the coast of the Hudson Bay Lowlands. In the 1960s, fewer than a hundred caribou were estimated for the population north of the Nelson River (C. Jonkel, S. Kearney, pers. comm.) and sparse groups of <50 animals were counted further south (Abraham and Thompson 1998). Caribou numbers have been increasing steadily (30- to 50-fold) since (Williams and Heard 1986; C. Jonkel, S. Kearney, and R. Brook, pers. comm.), while the animals are also expanding their summer range toward the coast (Abraham and Thompson 1998), thus increasing potential interactions with arriving bears (Fig. 3). Similarly, snow goose abundance has increased 5- to 20-fold across the region since the 1960s (Hanson et al. 1972; Kerbes et al. 2006; Alisauskas et al. 2011), with highest increase and geographic expansion being on the Cape Churchill Peninsula (Rockwell et al. 2009).

Although the scarcity of snow geese in the region during the 1960s likely explains their absence in Russell's study, it is important to note that considerably more (96.2%) of his bird remains were left unidentified compared to our study (21.6%). This may have been due, in part, to our use of more stringent bird identification techniques (see Material and Methods). However, all of Russell's unidentified bird remains comprised “trace to 5.0%” amounts by volume, whereas only 16.2% of our snow goose remains occurred in “trace to 5.0%” amounts. The remaining 83.8% of our scats with snow goose comprised an average of 65.0% of a scat pile by volume, with nearly 40% comprising >90% of a pile. Given the size of lesser snow geese and direct observations of how polar bears consume them (Iles et al. 2013), these larger volumes seem reasonable and their absence in Russell's study further suggest that the unidentified bird remains were likely not snow geese.

Polar bears seem to have taken advantage of the substantial increase in availability of both caribou and snow geese (Table 1). During the summer months, when the two species are raising their offspring, polar bears arriving onshore now regularly overlap herds of caribou and flocks of geese as the bears travel along the coast and move inland (Iles et al. 2013; L. J. Gormezano and R. F. Rockwell, unpubl. obs.). The increased co-occurrence of polar bears and the now plentiful caribou and snow geese facilitate opportunities for both predation as well as scavenging of kills made by other predators (e.g., wolves, *Canis lupus*, [Brook and Richardson 2002]; grizzly bears, *Ursus arctos*, [Rockwell et al. 2008]). Predation events on other waterfowl species during incubation or brood rearing on our study area (Table 1) and elsewhere (e.g., Madsen et al. 1998; Drent and Prop 2008) suggest that other avian species are similarly vulnerable.

Egg remains occurred in 4.6% of scats we collected along the coast, contrasting with Russell's study that reported no eggs (Russell 1975). Earlier observations had documented polar bears eating eggs as part of a varied diet (e.g., Harrington 1965; Pedersen 1966) or had reported them in the stomachs of harvested bears (pers. comm. to R. H. Russell 1975). Russell (1975) found egg remains in 5.0% of scats on the Twin Islands in James Bay, Ontario, but concluded that foraging on eggs was likely uncommon because polar bears were on the ice during the peak periods of hatch. With breakup occurring on average 22 days earlier, however, polar bears are arriving onshore sooner and are overlapping the incubation period of snow geese, common eiders, and other species of waterfowl (Rockwell and Gormezano 2009). Reports of polar bears consuming eggs of nesting waterfowl have increased across the polar bears' range (see Drent and Prop 2008; Smith et al. 2010). We also observed polar bears capturing adult birds (e.g., snow geese, Canada geese, common eiders) guarding their nests in addition to consuming their eggs. Consistent with our observations, we found that 25% of the scats with egg remains occurred in the same pile as the remains of adult snow geese.

Although the overall proportion of mammals in our scats has not changed substantially from Russell's study (24.6% vs. 32.1% of scats, respectively), we found caribou (above), more polar bear remains, and fewer rodent remains in our samples (Table 2). Assuming the rodent estimates in Russell (1975) are typical for the Cape Churchill area, the difference in rodents may be due either to our sampling occurring during 3- to 5-year cyclic fluctuations (Krebs and Myers 1974) or to declines in peak lemming abundance thought to be associated with warmer temperatures during fall freeze-up and subsequent high levels of precipitation into early winter that drive lemmings to higher ground where they are less protected through the harsh winter (Scott 1993).

The increased number of scats with polar bear remains relative to the 1960s (Table 2) is consistent with reported higher rates of cannibalism (i.e., intraspecific predation and/or scavenging). Several authors have speculated that because of earlier breakup of ice, nutritional stress could lead to increased intraspecific aggression and cannibalism (e.g., Taylor et al. 1985; Amstrup et al. 2006; Stirling et al. 2008). Recent observations of intraspecific attacks initiated by polar bears in poor condition support this suggestion (Lunn and Stenhouse 1985; Taylor et al. 1985), but many instances of healthy polar bears initiating similar attacks have also been reported (Taylor et al. 1985; Derocher and Wiig 1999; Dyck and Daley 2001; Stirling and Ross 2011). Furthermore, not all polar bears that are killed are consumed, suggesting that there may be other reasons for this behavior (Taylor et al. 1985; Derocher and Wiig 1999).

Different types of vegetation, particularly grasses and marine algae, were pervasive; occurring in 84.9% of polar bear scat piles and this is similar to observations across the circumpolar range of polar bears (Koettlitz 1898; Pedersen 1966; Russell 1975). Although the overall proportion of vegetation items has not changed since the 1960s (67% and 69%), the proportion of Lyme grass has increased while other grasses have decreased (Table 2). Like other predatory mammals, polar bears might consume vegetative roughage (e.g., grass stalks, marine algae, moss) as part of self-medicative efforts to reduce loads of worm parasites (Huffman 2003), to acquire a source of fiber to facilitate bowel movement (McKeown 1996) or to acquire nutrients that are lacking from animal sources. For example, polar bears preferentially consume the spikes of Lyme grass (Russell 1975; Lunn and Stirling 1985) that have protein-rich seed heads in early July through late August (Johnson 1987). Lyme grass has occurred along the entire coast of western Hudson Bay for many years (Jefferies et al. 2006) and unless polar bears are recently targeting it to fulfill a protein or other dietary need we can offer no firm explanation of its increased consumption. However, preliminary analyses of plant phenology on the Cape Churchill Peninsula (C. P. H. Mulder and R. F. Rockwell, unpubl. ms.) suggest that flowering and seed set is advancing although not as fast as sea ice dissolution. It is thus possible that polar bears are increasingly overlapping the seed heads much as snow goose eggs.

We also found a higher proportion of scats with mushrooms along the coastal portions of our study area than Russell (1975) found in the 1960s (Table 2). The two species we identified, *Lycoperdon pyriforme* and *L. perlatum*, occur from July through November along the entire western Hudson Bay coastline and thrive on driftwood that litters the coastline, fallen trees further inland, and soil substrates across the landscape (McKnight and McKnight 1998). Although Russell (1975) commented that mushrooms were typically found in low volumes (5–10%) with crowberries and suggested that they were consumed together at the same site, we found no patterns of co-occurrence of mushrooms with any other foods. Mushrooms were typically found in volumes of 10% or less, but we also found many (28.1% of scats with mushrooms) where mushrooms comprised 50% or more of a scat pile. There were four scats that contained only mushrooms, indicating that polar bears may consume them in large quantities when available, perhaps in attempt to acquire limiting micronutrients (e.g., Iversen et al. 2013).

The decrease in proportion of garbage in scats in the current diet may be due to marked changes in the availability of garbage both near the town of Churchill and in areas further east along the Hudson Bay coast. In 2005, the town of Churchill closed the landfill, which previously attracted numerous polar bears (Lunn and Stirling 1985). Garbage was subsequently secured from bears prior to recycling or removal from the area. Also, rules governing the securing and removal of waste from research camps, including Nester 1, from which Russell's Cape Churchill collections were based, became more stringent with the establishment of Wapusk National Park in 1996 (R. F. Rockwell, pers. obs.). Stored garbage depots were systematically removed from areas south of Cape Churchill and more effectively secured from polar bears in subsequent field seasons.

### General considerations

Our data indicate that polar bears are now foraging on increasingly abundant terrestrial prey such as caribou and snow geese and utilizing novel resources like eggs that have become newly available through climate-induced shifts in their onshore arrival. These observations combined with those of other studies and the diverse patterns of different foods in scats (Gormezano and Rockwell, in review) suggest that some polar bears are opportunistic omnivores. If this observed foraging renders some present or future benefit, it may be an example of “diet mixing” (ingestion of multiple species over an animal's lifetime or life cycle that differ qualitatively to the consumer) (Singer and Bernays 2003), a foraging strategy shared by many predators in Arctic ecosystems (Samelius and Alisauskas 1999; Elmhagen et al. 2000). This mode of foraging is similar to that observed in other bear species that are known to shift their diet regularly to exploit both seasonally (e.g., Persson et al. 2001) and newly available resources (Beckmann and Berger 2003) to meet their nutritional needs. In the closely related brown bear, dramatic differences in diet have been observed in response to local prey and vegetation abundance (e.g., Hilderbrand et al. 1999), competition (e.g., Gende and Quinn 2004), and environmental change (e.g., Rodríguez et al. 2007).

It is generally agreed that polar bears diverged from brown bears at least 600,000 years ago and evolved to survive in the specialized Arctic environment (Hailer et al. 2012; Cahill et al. 2013; Weber et al. 2013). One or more hybridization events have likely occurred since then, evidenced by brown bear mitochondrial DNA having introgressed into polar bear lines (Hailer et al. 2012). It has been suggested that such events may have helped polar bears persist through multiple interglacial warm phases (Edwards et al. 2011; Hailer et al. 2012). We suggest that the wide range of foraging behaviors observed for polar bears, like those present in brown bears, may reflect an inherent plasticity and shared genetic legacy that was likely retained over time (e.g., Agosta and Klemens 2008; Miller et al. 2012; Weber et al. 2013). Among those polar bears foraging, the shifts in the diet that have occurred (and are occurring) since Russell's (1975) study may be an innate plastic response to changing prey availability and exemplify the type of foraging behavior that these polar bears are capable of as climate change reduces their opportunities to hunt seals. Pending the outcome of current genetic analyses, however, it is yet unclear how many polar bears are exhibiting this behavior and thus the extent of any benefits that may be gleaned from it.

There is evidence that body mass and survival of at least some demographic classes of polar bears has declined coincident with the advancing date of breakup of Hudson Bay sea ice (e.g., Stirling and Parkinson 2006; Regehr et al. 2007). It is suggested that the declines are the result of the bears becoming increasingly nutritionally stressed and that this may, in turn, lead them to seek alternative food sources (Stirling and Parkinson 2006; Regehr et al. 2007). While possible, this seems unlikely to be the only cause of such terrestrial foraging because land-based hunting, scavenging, and grazing actually predate recorded climate-related changes (e.g., Pedersen 1966; Russell 1975).

Also, polar bears have switched between major prey items in the past when nutritional stress was likely not a causative factor. For example, Thiemann et al. (2008) found that polar bears switched their primary consumption from bearded to ringed seals when the abundance of the two species changed in western Hudson Bay. The switch was independent of the date of ice breakup and they concluded that polar bears are “… capable of opportunistically altering their foraging to take advantage of locally abundant prey, or to some degree compensating for a decline in a dominant prey species.” (Thiemann et al. 2008). Our observations on consumption of increasingly abundant caribou, snow geese, and their eggs are consistent with this assessment. Observations of polar bears coming ashore seeking eggs even while seals were still available on the ice (Madsen et al. 1998; Drent and Prop 2008) lend additional support to their prey switching abilities and general plasticity in foraging.

Current threats to the persistence of polar bears in western Hudson Bay are clear as the ice-free season expands, limiting polar bear access to seals on the ice (e.g., Stirling and Derocher 2012). However, with a history of adaptive foraging behavior and pursuit of novel prey across their Arctic habitat (e.g., Dyck and Romberg 2007; Smith et al. 2010), it is unlikely that polar bears will abstain from exploiting new terrestrial resources solely because they were ignored in the past in favor of more easily accessible marine prey. Some polar bears currently eat a variety of terrestrial animals and plants during the ice-free period, taking opportunistic advantage of abundant species. We suggest that research now focus on determining both the number of polar bears making this shift and the nutritional and energetic gains associated with this shifting terrestrial diet. Furthermore, these gains must be considered when modeling future polar bear survival. Shifts in diet composition, even for what may comprise a small fraction of the annual nutritional and energy budget may become increasingly important for some individuals in the population as ice conditions worsen (e.g., Dyck and Kebreab 2009; Rockwell and Gormezano 2009).
